# Polymorphisms of Killer Ig-like Receptors and the Risk of Glioblastoma

**DOI:** 10.3390/jcm12144780

**Published:** 2023-07-19

**Authors:** Haeyoun Choi, In-Cheol Baek, Soon A Park, Jae-Sung Park, Sin-Soo Jeun, Tai-Gyu Kim, Stephen Ahn

**Affiliations:** 1 Department of Microbiology, College of Medicine, The Catholic University of Korea, Seoul 06591, Republic of Korea; chlgosl@catholic.ac.kr (H.C.);; 2 Catholic Hematopoietic Stem Cell Bank, College of Medicine, The Catholic University of Korea, Seoul 06591, Republic of Korea; 3 Department of Biomedicine and Health Sciences, College of Medicine, The Catholic University of Korea, Seoul 06591, Republic of Korea; 4 Department of Neurosurgery, Seoul St. Mary’s Hospital, College of Medicine, The Catholic University of Korea, Seoul 06591, Republic of Korea

**Keywords:** KIR, polymorphism, glioblastoma, immunogenetics, Koreans

## Abstract

Purpose: The immune responses of natural killer (NK) cells against cancer cells vary by patient. Killer Ig-like receptors (KIRs), which are some of the major receptors involved in regulating NK cell activity for killing cancer cells, have significant genetic variation. Numerous studies have suggested a potential association between the genetic variation of KIR genes and the risk of development or prognosis of various cancer types. However, an association between genetic variations of KIR genes and glioblastoma (GB) remains uncertain. We sought to evaluate the association of genetic variations of KIRs and their ligand genes with the risk of GB development in Koreans. Methods: A case–control study was performed to identify the odds ratios (ORs) of KIR genes and Classes A, B, and, C of the human leukocyte antigen (HLA) for GB. The GB group was comprised of 77 patients with newly diagnosed IDH-wildtype GB at our institution, and the control group consisted of 200 healthy Korean volunteers. Results: There was no significant difference in the frequency of KIR genes and KIR haplotypes between the GB and control groups. Genetic variations of KIR-2DL1, 3DL1, and 3DS1 with their ligand genes (HLA-C2, HLA-Bw4/6, and Bw4, respectively) had effects on the risk of GB in Korean patients. The frequency of KIR-2DL1 with HLA-C2 (OR 2.05, CI 1.19–3.52, *p* = 0.009), the frequency of KIR-3DL1 without HLA-Bw4 (80I) (OR 8.36, CI 4.06–17.18, *p* < 0.001), and the frequency of KIR-3DL1 with Bw6 (OR 4.54, CI 2.55–8.09, *p* < 0.001) in the GB group were higher than in the control group. In addition, the frequency of KIR-2DL1 without HLA-C2 (OR 0.44, CI 0.26–0.75, *p* = 0.003), the frequency of KIR-3DL1 with HLA-Bw4 (80T) (OR 0.13, CI 0.06–0.27, *p* < 0.001), the frequency of KIR-3DL1 without Bw6 (OR 0.27, CI 0.15–0.49, *p* < 0.001), and the frequency of KIR-3DS1 with Bw4 (80I) (OR 0.03, CI 0.00–0.50, *p* < 0.001) in the GB group were lower than in the control group. Conclusions: This study suggests that genetic variations of KIRs and their ligand genes may affect GB development in the Korean population. Further investigations are needed to demonstrate the different immune responses for GB cells according to genetic variations of KIR genes and their ligand genes.

## 1. Introduction

Glioblastoma (GB) is the most common primary brain malignancy and has a devastating prognosis [1,2]. The median survival is less than 2 years, despite the aggressive multimodal treatments, which include maximal surgical resection and concomitant chemoradiation, followed by adjuvant temozolomide chemotherapy [3]. In the era of immunotherapy, various immunotherapeutic approaches have been explored to overcome the fatal prognosis of GB; however, the clinical outcomes are inconclusive [4,5,6]. The heterogeneous immune responses of cancer cells among patients and the immunosuppressive tumor microenvironment associated with GB have been suggested as major obstacles impeding the success of immunotherapy for GB [4,5,6,7,8].

Natural killer (NK) cells, which are among the major immune cell types that can recognize and kill cancer cells, play a key role in the host’s antitumor immune response, thereby impacting glioma formation and progression [9,10]. Due to their ability to kill cancer cells, the adoptive transfer of NK cells has been most frequently explored as a novel method for cancer treatment [9,10,11]. However, the tumor microenvironment of GB is known to be severely immunosuppressive and to involve low infiltration of lymphocytes [12]. Tumor-associated macrophages (TAMs) and myeloid-derived suppressor cells (MDSCs) are considered important due to their dominant proportion and correlation with a poor prognosis, respectively [12,13,14]. Several studies have demonstrated NK cell infiltration in GB, and their recruitment to brain tumors is known to be mediated by the CX3CL1–CX3CR1 axis [15,16]. Once in the GB microenvironment, NK cells encounter high levels of inhibitory molecules, including MHC class I molecules, PD-L1, and lectin-like transcript-1 (LLT1) [17,18,19,20,21,22]. In addition, secretory molecules, such as regeneration and tolerance factor (RTF), suppressive cytokines, hypoxia, and a high concentration of lactate impair NK cell function [23,24,25,26,27,28,29].

The activity of NK cells in targeting cancer cells is regulated by the balance of activating and inhibitory signals from their various receptors [30,31]. Among these receptors, killer immunoglobulin-like receptors (KIR), which recognize their human leukocyte antigen (HLA) I class ligands, play a major role in regulating NK cell activity for killing cancer cells [32]. Each KIR can transduce either activating or inhibitory signals through its cytoplasmic domain immunoreceptor tyrosine-based activation motifs (ITAM; YXX-(L/I/V)-X_6-8_-YXX-(L/I/V)) or immunoreceptor tyrosine-based inhibitory motifs (ITIM; (I/V/L/S)-XYXX-(L/V))), respectively [33,34]. KIR genes, which are encoded on chromosome 19q13.4, comprise a gene family that shows extensive polymorphisms and includes 16 highly homologous and closely related genes (eight inhibitory genes, six activating genes, and two pseudogenes) [35]. Different combinations of these KIR genes create two major groups of KIR haplotypes: the Group A haplotype and Group B haplotype. Furthermore, three distinct genotype groups—AA (homozygote), BB (homozygote), and AB (heterozygote)—exist according to the haplotypes of KIR [36].

Numerous studies have found potential associations between polymorphisms of KIR genes and various cancer types [37,38,39,40]; however, only a few studies have investigated associations between KIR genes and an increased risk of developing GB [40,41]. One recent study that included 31 GB patients suggested that the KIR gene 2DL3 was related to an increased risk of GB, and another study of 108 GB patients suggested that the KIR gene 2DS4*00101 was related to good prognosis in GB patients [40]. In this study, we evaluated whether the frequencies of KIR genes and their ligand genes were different between a GB group and a healthy control group in Koreans. To the best of our knowledge, this is the first study to suggest a higher frequency of KIR2DL1 with HLA-C2 in a GB group.

## 2. Materials and Methods

### 2.1. Glioblastoma and Control Groups

This study was approved by the Institutional Review Board (IRB) of Seoul St. Mary’s Hospital (KC18TESI0024). Patients with newly diagnosed GB who were present for follow-up at the Neuro-oncology Center of Seoul St. Mary’s Hospital from March 2018 March to December 2019 were included as the patient group after providing informed consent. We only included newly diagnosed primary IDH-wildtype GB and excluded secondary or recurrent GB according to the 2021 World Health Organization Classification of Tumors of the Central Nervous System (CNS). The IDH 1 mutation was evaluated via immunohistochemistry or directing sequencing, and 1p/19q co-deletion was detected by using fluorescence in situ hybridization (FISH). The O [6]-Methylgua-nine-DNA methyltransferase (MGMT) gene methylation status was evaluated by using polymerase chain reaction (PCR). Patients with a previous history of other cancers or autoimmune diseases were excluded. Details of the methods of collecting clinical variables were described in a previous report [42].

The control group consisted of 200 healthy Korean volunteers who were genetically unrelated to one another. The mean age of the control group was 30 years, and the proportion of males was 51.0% (102 males and 98 females). All experiments were performed with the approval of the IRB.

### 2.2. DNA Extraction and HLA Genotyping

After we received informed consent from the patients, we acquired 4 mL of peripheral blood for DNA extraction. Peripheral blood mixed with ethylenediaminetetraacetic acid (EDTA) and genomic DNA was extracted according to standard methods by using TIANamp Genomic DNA Extraction Kits (Tiangen Biotech Corporation, Beijing, China) according to the manufacturer’s instructions.

The genotyping of KIR genes was performed by using polymerase chain reaction with sequence-specific primers (PCR-SSP), as described in a previous study [39]. We performed specific amplifications of the KIR genes with 50 forward and reverse primers. PCR was carried out with primers in a reaction volume of 10 mL containing a buffer (50 mM KCl, 10 mM TriseHCl pH 9.0, 1.5 mM MgCl 2, 0.1% Triton X-100), 0.1–0.5 mM of each nucleotide primer, 2.5 mM dNTPs, 100 ng genomic DNA, and 0.25 U I-start Taq Polymerase (iNtRON Biotechnology, Seongnam, Republic of Korea). The amplifications were carried out in a My Cycler™ thermocycler (Bio-Rad, Hercules, CA, USA). A total of 35 cycles of PCR were completed by using the following steps: 25 s at 91 °C, 45 s at 65 °C, and 30 s at 72 °C (first 4 cycles); 25 s at 91 °C, 45 s at 60 °C, and 30 s at 72 °C (next 26 cycles); 25 s at 91 °C, 60 s at 55 °C, and 120 s at 72 °C (last 5 cycles); finally, a 10-min extension at 72 °C. The presence or absence of PCR products was determined following separation on a 2% agarose gel containing 0.5 mg/mL ethidium bromide.

The genotyping of HLA-A,-B, and -C was performed by using PCR-sequence-based typing (PCR-SBT) methods, as described in previous studies [42,43]. In total, 19 alleles for HLA-A, 38 alleles for HLA-B, and 22 alleles for HLA-C were detected. Amino acid sequences for HLA-A, -B, and -C with a resolution of 4 digits were obtained from the international ImMunoGeneTics references. Analysis of variant amino acids was performed across all HLA-A, -B, and -C alleles present in the genotyping results.

### 2.3. Statistical Analysis

All clinical variables were characterized in a descriptive manner. Kaplan–Meier survival analysis and the log-rank test were used to estimate the median overall survival (OS) and progression-free survival (PFS). The difference in frequencies of KIR alleles in the control and GB groups was compared by using the Chi-square test or Fisher’s exact test. The odds ratio (OR) and 95% confidence interval (CI) were estimated by using logistic regression. Statistical analysis was performed with RStudio, version 1.4.1717. *p*-values < 0.05 were considered statistically significant.

## 3. Results

### 3.1. Clinical Characteristics of the GB Group

The clinical characteristics of the patients in the GB group who met the eligibility criteria are presented in Table 1. Among the total 77 patients, 43 (53.8%) were male. The median age of the GB group was 62.5 (range: 20–81) years. There were only seven patients with an unknown IDH mutation and/or 1p19q co-deletion. The median overall survival of the GB group was 18.3 months (range: 4–87 months).

### 3.2. Associations of Genotypes and Haplotypes of KIR Genes with GB Risk

When we classified genomic data into A and Bx haplotypes, there was no significant difference in frequency between the two groups (A: 51.9% vs. 56.0%, *p* = 0.369, Bx: 48.1% vs. 44.0, *p* = 0.712). Additionally, there was no difference in KIR genotypes according to the Allele Frequency Net Database (AFND; 2020). The detailed distribution of genotypes and haplotypes of KIR genes between the two groups is illustrated in Supplementary Figure S1. Furthermore, there was no significant difference in the frequency of KIR genes between the GB and control groups. However, in the analysis of the 2DL5, 2DS4, and 3DP1 genes, we observed a significantly higher frequency of the BB subtype of 2DL5 in the GB group compared to that in the control group (23.3% versus 6.8%, *p* = 0.023, OR 4.20, CI 1.21–14.53). The detailed distribution of the KIR genes is presented in Table 2, and the detailed subtype distribution of the 2DL5, 2DS4, and 3DP1 genes is presented in Supplementary Table S1.

### 3.3. Associations of KIR Genes and Their Ligand Genes with GB Risk

Next, we evaluated the associations of KIR genes and their ligand genes in relation to GB risk. To validate the combinations of KIR receptors and their ligands, as reported previously [44], we analyzed the following pairs: (1) KIR-2DL1 and HLA-C2, (2) KIR-2DL2/3 and HLA-C1, (3) KIR-3DL1 and HLA-Bw4 (80I or 80T)/Bw6, (4) KIR-3DL2 and HLA-A3 or A11, (5) KIR-2DS1 and HLA-C2, (6) KIR-2DS2 and HLA-C1, and (7) KIR-3DS1 and HLA-Bw4 (80I or 80T). Among them, we observed significantly higher frequencies of KIR-2DL1with HLA-C2 (OR 2.05, CI 1.19–3.52, *p* = 0.009), KIR-3DL1 without HLA-Bw4 (80I) (OR 8.36, CI 4.06–17.18, *p* < 0.001), and KIR-3DL1 with Bw6 (OR 4.54, CI 2.55–8.09, *p* < 0.001) in the GB group compared to the control group. Additionally, we observed significantly lower frequencies of KIR-2DL1 without HLA-C2 (OR 0.44, CI 0.26–0.75, *p* = 0.003), KIR-3DL1 with HLA-Bw4 (80T) (OR 0.13, CI 0.06–0.27, *p* < 0.001), KIR-3DL1 without Bw6 (OR 0.27, CI 0.15–0.49, *p* < 0.001), and KIR-3DS1 with Bw4 (80I) (OR 0.03, CI 0.00–0.50, *p* < 0.001) in the GB group compared to the control group. The detailed distribution of KIR genes and their ligand genes between the two groups is presented in Table 3.

## 4. Discussion

NK cells, which are known to kill cancer cells, are some of the most commonly investigated immune cells in immuno-oncology [10,11]. Mature NK cells are large granular lymphocytes that are defined as non-T, non-B lymphocytes. They lack CD3, CD5, CD19, and CD20, and they comprise about 5–15% of lymphocytes in human peripheral blood [45,46]. When stimulated, NK cells can produce interferon gamma (IFN-γ) and exhibit cellular cytotoxicity [45]. NK cells can be further grouped into CD56 bright conventional NK cells (cNK), which display immunoregulatory functions, and CD56 dim cNK cells, which have cytotoxic functions [45,46,47,48]. Recently, several studies have suggested that genetic variations among NK cells may be associated with the risk and/or prognosis of cancer in patients [37,38,39,40]. NK cells are remarkably diverse, displaying up to 30,000 phenotypic populations within one individual [49]. The diversity of NK cells and the differential expression of surface receptors are affected by several factors, such as genotype, epigenetic regulation, environment, and NK cell education [49,50]. This diversity is closely linked to NK cell function because NK cell activation is controlled by the signals from activating and inhibitory receptors expressed on the cell surface. In addition, when the infiltration of NK cells in various cancer types was analyzed, activating-receptor-positive NK cells, such as NKp30+ or NKp46+ cells, were associated with favorable survival [51]. Particularly, the expression of NKp46, Nkp44, and NKp30 was shown to be crucial in NK-cell-mediated lysis of glioblastoma tumor cells [52]. This indicated that the clonality and phenotypic characteristics of tumor-infiltrating NK cells could affect the prognosis and survival in cancer patients.

The expression of KIR genes, which are some of the main receptors of NK cells used to control activating or inhibitory responses toward target cells, also shows diversity, although the exact mechanism remains to be elucidated. For example, NK cells from 7% of individuals with the KIR2DL1 gene did not express the corresponding receptor, and 15% of individuals with the KIR3DL1 gene also did not show the corresponding receptor expression on NK cells. Moreover, KIR2DL3 was preferentially expressed when both the KIR2DL2 and KIR2DL3 genes were present [53]. The expression of KIR genes has been widely investigated in various cancers, including lung, colorectal, and gastric cancers [37,38,39,54]. However, a few studies have tried to identify the effects of genetic variations of KIR genes on the risk of brain cancers [40,41]. A recent study comparing 31 Turkish GB patients with the 2DL3 allele and 50 control individuals who did not carry the KIR-2DL3 allele suggested that KIR-2DL3 may be related to the risk of GB (77.4% vs. 25.5%, *p* = 0.002) [40]. Another study that included 108 Nordic patients with GB suggested that the KIR-2DS4*00101 gene could provide a better prognosis in GB patients (HR 0.6, *p* = 0.034) [41].

In this study, we analyzed the frequencies of KIR genes in 77 Korean GB patients with IDH-wildtype and compared them with those of 200 healthy Koreans. We also analyzed the frequencies of KIR ligand genes, including HLA-type A3, A11, Bw4, Bw6, C1, or C2. While there was no significant difference in the frequency of KIR genes between the GB and control groups, the genetic variations of KIR-2DL1, 3DL1, 3DS1, and their corresponding ligand genes (HLA-C2, HLA-Bw4/6, and Bw4, respectively) had an effect on the risk of GB in Korean patients. The biological mechanisms linking polymorphisms of KIRs and their ligand genes to cancer risk are multifactorial. The presence or absence of certain KIR genes and their ligands can influence the activating or inhibitory receptor functions of NK cells or other immune cells. Additionally, the binding intensity between KIR receptors and their ligands can alter the signals that regulate NK cell activity. Specifically, the increased risk of GB in the patient group with KIR-2DL1 and HLA-C2 may be explained by the inhibitory nature of KIR-2DL1, as it transmits inhibitory signals via its ITIMs-containing cytoplasmic domain when bound to HLA-C2. This inhibitory signaling suppresses the antitumor effect of NK cells against HLA-C2-expressing GB [30,31,32]. Moreover, genetic interactions between KIR genes and other immune-related genes, such as cytokines or HLA genes, may further influence the GB risk.

Adoptive cell transfer by using immune cells has been extensively investigated for various cancers, including GB [55,56,57]. However, the clinical results are inconclusive and limited [55,56,57]. One of the major reasons suggested for the unsatisfactory response is the heterogeneity in the immune response among patients [4,5,6,56]. As there are several genetic variations of immune-related genes, including HLA and KIR genes, the immune responses vary among patients [37,38,39,40,42,43]. In this context, analysis and identification of genetic variations among cancer patients are crucial [37,38,39,40,42].

To the best of our knowledge, our study is the first to analyze polymorphisms of KIR genes and the risk of brain malignancies in Koreans. Considering ethnic differences in the distribution of KIR genes and that Korean populations have lower frequencies of 2DL2 and 2DS2 genes compared to those in Western groups [58,59], our results may provide evidence to help understand the heterogeneous immune response between NK cells and GB cells among patients. Given the recent advancements that have elucidated the distinct pathogenesis of primary and secondary GB transformed from low-grade glioma, which mostly presented IDH mutations, the impact of KIR gene polymorphism on the risk of secondary GB could differ from our results [60,61]. As GB with the IDH-mutant has a distinct clinical prognosis from that of GB with the IDH-wildtype, inclusion of GB with the IDH-mutant can produce biased results [62,63]. Our study included a homogeneous disease entity of GB with the IDH-wildtype that was diagnosed by using the 2021 WHO classification [61], while other previous studies might have included GB with IDH-mutant or anaplastic oligodendroglioma with similar histological features of GB [40,41]. Further investigation is needed to explore the association between polymorphisms in immune-related genes and secondary GB. Taken together, our findings may also help in understanding the role of immune cells with KIR receptors, such as NK cells, in the tumorigenesis and progression of GB. Additionally, they provide essential information on NK-cell-based therapy for glioblastoma in Koreans, offering a foundation for further research and potential therapeutic strategies.

Our study has several limitations. First, we did not adjust for all potential factors that could influence hazard ratios, such as sex, age, and several lifestyle factors. Second, the possibility of linkage disequilibrium with other unmeasured alleles in the region in GB development cannot be excluded. Third, the expression or biological function of KIR genes on NK cells should be further evaluated with in vitro experiments. Fourth, given the retrospective design of our study, it was not possible for us to exclude the potential influence of previous viral infections that may have stimulated NK cells, despite the exclusion of any clinical history of autoimmune or hematologic disorders. Further investigations to elucidate the different immune responses according to genetic variations of KIR genes and their ligand genes are needed. Fifth, we did not perform a power analysis. The sample size was determined based on the feasibility and availability of participants during the data collection period. Lastly, the survival outcomes in groups with genetic variations of KIR genes and their ligands that affect the risk of GB did not differ from those of other groups in our study. Further investigations are needed to analyze the survival outcomes according to genetic variations of KIR genes and their ligand genes. 

## 5. Conclusions

Genetic variations of KIR genes and their ligand genes may affect GB development in Korean populations, which may provide a foundation for NK-cell-based immunotherapy for GB patients. Further studies are needed to demonstrate the different immune responses of NK cells against GB cells according to the genetic variations of KIR genes and their ligand genes. In addition, a comprehensive study providing the relationship of the KIR genotype and phenotype with NK cell function could greatly enhance the understanding of the importance of KIR in the development of GB.

## Figures and Tables

**Table 1:** Clinical characteristics of the GB group.

Characteristics	GB Group (*n* = 77)
Male sex, *n* (%)	43 (55.8%)
Median age, years (range)	62.5 (20–81)
IDH1 mutation, *n* (%)	
Yes	0 (0%)
No	70 (90.9%)
Unknown	7 (9.1%)
1p/19q co-deletion, *n* (%)	
Yes	0 (0%)
No	70 (90.9%)
Unknown	7 (9.1%)
MGMT methylation, *n* (%)	
Yes	40 (51.9%)
No	37 (48.1%)
Unknown	0 (0%)
Median progression-free survival, months (range)	8.7 (2–87)
Median overall survival, months (range)	18.3 (4–87)

**Table 2:** Distribution of the KIR genes between the GB and control groups.

Function	Genes		GB (*N* = 77) *n* (%)		Control (*N* = 200) *n* (%)		*p*-Value
**Inhibitory**							
	2DL	1	77	(100.0)	200	(100.0)	>0.999
		2	15	(19.5)	25	(12.5)	0.139
		3	77	(100.0)	199	(99.5)	>0.999
		4	77	(100.0)	200	(100.0)	>0.999
		5	30	(39.0)	74	(37.0)	0.762
	3DL	1	75	(97.4)	183	(91.5)	0.111
		2	77	(100.0)	200	(100.0)	>0.999
		3	77	(100.0)	200	(100.0)	>0.999
**Activating**							
	2DS	1	27	(35.1)	72	(36.0)	0.774
		2	18	(23.4)	31	(15.5)	0.124
		3	10	(13.0)	37	(18.5)	0.273
		4	75	(97.4)	183	(91.5)	0.111
		5	22	(28.6)	48	(24.0)	0.433
	3DS	1	24	(31.2)	73	(36.5)	0.694
**Pseudogene**							
	2DP	1	77	(100.0)	200	(100.0)	>0.999
	3DP	1	77	(100.0)	200	(100.0)	>0.999

**Table 3:** The association of KIR genes and their ligands between the GB and control groups.

KIR Genes	Ligand Genes (HLA)	GB (*N* = 77) *n* (%)		Control (*N* = 200) *n* (%)		*p*-Value	Odds Ratio (95% CI)
2DL1+	C2+	36	(46.8)	60	(30.5)	0.009	2.05 (1.19–3.52)
	C2−	39	(50.6)	140	(70.0)	0.003	0.44 (0.26–0.75)
2DL2+	C1+	14	(18.2)	25	(12.5)	0.148	
	C1−	1	(1.3)	0	(0.0)	0.278	
2DL3+	C1+	74	(96.1)	185	(92.5)	0.276	
	C1−	3	(3.9)	7	(3.5)	0.874	
3DL1+	Bw4+	62	(80.5)	141	(70.5)	0.091	
	Bw4−	13	(16.9)	42	(21.0)	0.442	
	Bw4 (80I)+	8	(10.4)	94	(47.0)	<0.001	0.13 (0.06–0.27)
	Bw4 (80I)−	67	(87.0)	89	(44.5)	<0.001	8.36 (4.06–17.18)
	Bw4 (80T)+	20	(36.0)	33	(16.5)	0.073	
	Bw4 (80T)−	55	(71.4)	150	(75.0)	0.998	
	Bw6+	56	(72.7)	74	(37.0)	<0.001	4.54 (2.55–8.09)
	Bw6−	19	(24.7)	109	(54.5)	<0.001	0.27 (0.15–0.49)
3DL2+	A3+ or A11+	20	(26.0)	47	(23.5)	0.666	
	A3− and A11−	57	(74.0)	153	(76.5)	0.666	
2DS1+	C2+	13	(16.9)	23	(11.5)	0.233	
	C2−	14	(18.2)	49	(24.5)	151	
2DS2+	C1+	17	(22.1)	31	(15.5)	0.195	
	C1−	1	(!.3)	0	(0.0)	0.106	
3DS1+	Bw4+	18	(23.4)	60	(30.0)	0.272	
	Bw4−	6	(7.8)	13	(6.5)	0.703	
	Bw4 (80I)+	0	(0.0)	35	(17.5)	<0.001	0.03 (0.00–0.50)
	Bw4 (80I)−	24	(31.2)	38	(19.0)	0.379	
	Bw4 (80T)+	5	(6.5)	13	(6.5)	0.998	
	Bw4 (80T)−	19	(24.7)	60	(30.0)	0.379	

## Data Availability

Data are available on request due to privacy/ethical restrictions.

## Supplementary Materials

The following supporting information can be downloaded at: https://www.mdpi.com/article/10.3390/jcm12144780/s1, Supplementary Figure S1. Distribution of KIR haplotypes and genotypes between GB and control groups. Table S1: Distribution of the 2DL5, 2DS4, and 3DP1 gene subtypes between the GB and control groups. Table S2: Distribution of the HLA-A-type genes between the GB and control groups. Table S3: Distribution of the HLA-B-type genes between the GB and control groups. Table S4: Distribution of the HLA-C-type genes between the GB and control groups.
