# Supplementary material for: Polymorphisms of Killer Ig-like Receptors and the Risk of Glioblastoma
Source: J Clin Med. 2023 Jul 19;12(14):4780. doi: 10.3390/jcm12144780 (PMC10380963; doi:10.3390/jcm12144780)
Supplement: Supplementary file 1 [file jcm-12-04780-s001.zip › jcm-2471191-supplementary.pdf]

Supplementary Table S1. Distribution of the 2DL5, 2DS4, and 3DP1 gene subtypes between the GB and control groups.

Subtype	GB (N=77) n (%)		Control (N=200) n (%)		*P*-value	Odds ratio (95% CI)
**2DL5**						
AA	22	(73.3)	60	(81.1)	0.816	
BB	7	(23.3)	5	(6.8)	0.023	4.20 (1.21-14.53)
AB	1	(3.3)	9	(12.2)	0.293	
**2DS4**						
Del/Del	13	(7.3)	37	(20.2)	0.754	
Full/Full	37	(49.3)	95	(51.9)	0.934	
Del/Full	25	(33.3)	51	(27.9)	0.244	
**3DP1**						
Del/Del	65	(84.4)	181	(90.5)	0.150	
Full/Full	0	(0.0)	0	(0.0)	> 0.999	-
Del/Full	12	(15.6)	19	(9.5)	0.150	

Supplementary Table S2. Distribution of the HLA-A-type genes between the GB and control groups.

Group	Alleles	GB (N=77) n (%)		Control (N=200) n (%)		*P* value	Odds ratio (95% CI)
A1	01:01	5	(6.5)	5	(2.5)	0.110	-
A2	02:01	23	(29.9)	73	(36.5)	0.299	-
	02:06	15	(19.5)	35	(17.5)	0.701	-
	02:06	15	(19.5)	35	(17.5)	0.701	-
	02:07	7	(9.1)	16	(8.0)	0.768	-
A203	02:03	2	(2.6)	7	(3.5)	0.704	-
A3	03:01	4	(5.2)	7	(3.5)	0.518	-
A11	11:01	19	(24.7)	42	(21.0)	0.508	-
A24	24:02	28	(36.4)	75	(37.5)	0.861	-
A26	26:01	5	(6.5)	12	(6.0)	0.878	-
	26:02	2	(2.6)	18	(9.0)	0.065	-
	26:03	2	(2.6)	1	(0.5)	0.131	-
A29	29:01	2	(2.6)	1	(0.5)	0.131	-
A30	30:01	7	(9.1)	12	(6.0)	0.362	-
A31	31:01	7	(9.1)	21	(10.5)	0.727	-
A32	32:01	1	(1.3)	1	(0.5)	0.482	-
A33	33:03	22	(28.6)	53	(26.5)	0.728	-
	33:25	1	(1.3)	0	(0.0)	0.106	-
A68	68:01	1	(1.3)	0	(0.0)	0.106	-

Supplementary Table S3. Distribution of the HLA-B-type genes between the GB and control groups.

Group	Alleles	GB (N=77) n (%)		Control (N=200) n (%)		*P* value	Odds ratio (95% CI)
BW4	13:01	5	(6.5)	9	(4.5)	0.497	-
	38:02	2	(2.6)	7	(3.5)	0.704	-
	44:03	14	(18.2)	31	(15.5)	0.588	-
	52:01	7	(9.1)	9	(4.5)	0.142	-
	58:01	11	(14.3)	21	(10.5)	0.377	-
	27:04	0	(0.0)	1	(0.5)	0.534	-
	51:02	1	(1.3)	4	(2.0)	0.694	-
BW4 (80I)	24:02	28	(36.4)	75	(37.5)	0.861	-
	32:01	1	(1.3)	1	(0.5)	0.482	-
	51:01	8	(10.4)	37	(18.5)	0.101	-
	57:01	1	(1.3)	2	(1.0)	0.830	-
BW4 (80T)	13:02	8	(10.4)	14	(7.0)	0.350	-
	27:05	7	(9.1)	15	(7.5)	0.661	-
	37:01	5	(6.5)	4	(2.0)	0.059	-
	44:02	3	(3.9)	7	(3.5)	0.874	-
BW6	07:02	7	(9.1)	18	(9.0)	0.981	-
	08:01	1	(1.3)	2	(1.0)	0.830	-
	14:01	0	(0.0)	8	(4.0)	0.075	-
	15:01	20	(26.0)	34	(17.0)	0.091	-
	15:02	0	(0.0)	1	(0.5)	0.534	-
	15:07	0	(0.0)	3	(1.5)	0.280	-
	15:18	2	(2.6)	8	(4.0)	0.575	-
	27:05	7	(9.1)	15	(7.5)	0.661	-
	35:01	11	(14.3)	18	(9.0)	0.198	-
	40:01	4	(5.2)	13	(6.5)	0.685	-
	46:01	5	(6.5)	22	(11.0)	0.257	-

	51:01	8	(10.4)	37	(18.5)	0.101	-
	54:01	5	(6.5)	26	(13.0)	0.124	-
	81:01	0	(0.0)	1	(0.5)	0.534	-
	07:02	7	(9.1)	18	(9.0)	0.981	-
	08:01	1	(1.3)	2	(1.0)	0.830	-
	14:01	0	(0.0)	8	(4.0)	0.075	-

Supplementary Table S4. Distribution of the HLA-C-type genes between the GB and control groups.

Group	Alleles	GB (N=77) n (%)		Control (N=200) n (%)		*P* value	Odds ratio (95% CI)
C1	01:02	22	(28.6)	72	(36.0)	0.242	-
	01:03	0	(0.0)	3	(1.5)	0.563	-
	03:02	11	(14.3)	21	(10.5)	0.377	-
	03:03	14	(18.2)	36	(18.0)	0.972	-
	03:04	10	(13.0)	34	(17.0)	0.413	-
	07:01	1	(1.3)	0	(0.0)	0.106	-
	07:02	15	(19.5)	36	(18.0)	0.776	-
	07:04	2	(2.6)	8	(4.0)	0.575	-
	07:06	1	(1.3)	8	(4.0)	0.256	-
	08:01	4	(5.2)	30	(15.0)	0.012	0.3 (0.1 – 0.9)
	08:02	0	(0.0)	8	(4.0)	0.075	-
	08:03	0	(0.0)	3	(1.5)	0.280	-
	12:02	7	(9.1)	10	(5.0)	0.204	-
	12:03	1	(1.3)	3	(1.5)	0.900	-
	14:02	9	(11.7)	28	(14.0)	0.612	-
	14:03	11	(14.3)	24	(12.0)	0.608	-
C2	02:02	2	(2.6)	4	(2.0)	0.760	-
	04:01	16	(20.8)	22	(11.0)	0.034	2.1 (1.05 – 4.3)
	05:01	3	(3.9)	7	(3.5)	0.874	-
	06:02	12	(15.6)	19	(9.5)	0.150	-
	15:02	3	(3.9)	13	(6.5)	0.405	-
	15:05	2	(2.6)	2	(1.0)	0.318	-

Supplementary Figure S1. Distribution of KIR haplotypes and genotypes between GB and control groups.

Haplotype group	Genotype ID	3DL1	2DL1	2DL3	2DS4	2DL2	2DL5	3DS1	2DS1	2DS2	2DS3	2DS5	2DL4	3DL2	3DL3	2DP1	3DP1	GB N=77 n, (%)	Control N=200 n, (%)
AA	1	■	■	■	■								■	■	■	■	■	40(51.9)	111(55.5)
AA	180	■	■		■								■	■	■	■	■		1(0.5)
Bx	2	■	■	■	■		■	■	■			■	■	■	■	■	■	15(19.5)	27(13.5)
Bx	8	■	■	■	■		■	■	■		■		■	■	■	■	■	2(2.6)	19(9.5)
Bx	4	■	■	■	■	■				■			■	■	■	■	■	5(6.5)	9(4.5)
Bx	10	■	■	■	■					■			■	■	■	■	■	1(1.3)	4(2.0)
Bx	69		■	■			■	■	■			■	■	■	■	■	■		4(2.0)
Bx	75		■	■			■	■	■		■	■	■	■	■	■	■	1(1.3)	4(2.0)
Bx	3	■	■	■	■	■	■	■	■	■		■	■	■	■	■	■	4(5.2)	3(1.5)
Bx	70		■	■		■	■	■	■	■	■	■	■	■	■	■	■	1(1.3)	3(1.5)
Bx	6	■	■	■	■	■	■	■	■	■	■	■	■	■	■	■	■		2(1.0)
Bx	13	■	■	■	■	■	■	■		■	■		■	■	■	■	■	1(1.3)	2(1.0)
Bx	68		■	■		■	■	■	■	■		■	■	■	■	■	■		2(1.0)
Bx	159		■	■		■	■	■	■	■	■		■	■	■	■	■		2(1.0)
Bx	5	■	■	■	■	■	■			■	■		■	■	■	■	■		1(0.5)
Bx	11	■	■	■	■	■	■		■	■	■		■	■	■	■	■	2(2.6)	1(0.5)
Bx	16	■	■	■	■			■	■				■	■	■	■	■		1(0.5)
Bx	56	■	■	■	■		■	■	■	■	■	■	■	■	■	■	■		1(0.5)
Bx	166		■	■			■	■	■	■	■	■	■	■	■	■	■		1(0.5)
Bx	154		■	■	■		■	■	■			■	■	■	■	■	■		1(0.5)
Bx	331	■	■	■			■	■	■		■		■	■	■	■	■		1(0.5)
Bx	7	■	■	■	■	■	■	■	■	■	■		■	■	■	■	■		
Bx	9	■	■	■	■	■	■		■	■		■	■	■	■	■	■	1(1.3)	
Bx	29	■	■	■	■	■	■		■	■			■	■	■	■	■	1(1.3)	
Bx	36	■	■	■	■		■			■	■		■	■	■	■	■	2(2.6)	
Bx	72	■			■	■				■			■	■	■		■		
Bx	260	■			■	■				■			■	■	■		■	1(1.3)	
